# Acute Infective Periostitis in Children: Its Causation and Treatment

**Published:** 1894-02-03

**Authors:** A. H. Tubby

**Affiliations:** Surgeon to Out-Patients, Evelina Hospital for Sick Children


### Medical Progress and Hospital Clinics.

[*The Editor will be glad to receive offers of co-operation and contributions from members of the profession. All letters should be addressed to* The Editor, The Lodge, Porchester Square, London, W.]

### ACUTE INFECTIVE PERIOSTITIS IN CHILDREN: ITS CAUSATION AND TREATMENT.

By A. H. Tubby, M.S.Lond., F.R.C.S.Eng., Surgeon to Out-Patients, Evelina Hospital for Sick Children.

This disease is characterised by its sudden onset, often with typhoid-like symptoms, its rapid course, the early formation of pus, the frequent necrosis of a large part of the bone, and in many cases the complication of pyæmia.

The question why acute infective bone disease is so prone to occur in childhood must be left unanswered at present. It is sufficiently easy to speak of unstable tissue, tissues more or less embryonic, or in course of development. But is not the whole body in the same condition of rapid growth? Why the virus should attach itself more firmly to one growing tissue than another is still an open question, and demands future investigation and experiments.

If the tissues immediately on either side of the epiphysial line be examined, there will be found on the shaft side a region which presents the following appearances. It consists of two or three rows of cavities occupied by numerous osteoblasts and blood vessels, terminating in dilated capillary loops. The importance of this layer of embryonic, and therefore unstable, material between the newly-formed bone of the shaft and the epiphysial line, must not be underrated in the consideration of the origin of acute infective periostitis. We know, too, that in traumatic separation of the epiphyses the line of rupture passes directly through this zone, leaving the cartilage attached to the epiphysis. For brevity's sake, this region of active proliferation of cells and great vascularity will be called the juxta-epiphysial junction, and inflammation of it "juxta-epiphysitis."

With reference to the site of commencement of acute infective periostitis or acute infective necrosis—and by these terms I mean that disease due to the morbid action of the micrococcus pyogenes aureus, and not such bone inflammation as occurs after fevers or due to poisons—English authorities are agreed that it commences in the deeper layers of the periosteum. The Germans, however, hold that the primary focus of inflammation is in the medulla, and that the disease is really an acute osteomyelitis. But Ollier has shown that under favourable circumstances, removal of the periosteum alone does not give rise to necrosis, that a reproduction of periosteum from surrounding tissues occasionally follows, and that in animals even the simultaneous removal of both periosteum and marrow is not always followed by necrosis.

In very severe cases the shaft of the bone is found bare, and lying in a distinct cavity formed by the periosteum and the cap of epiphysial cartilage. The ends of the diaphysis are often eroded and gangrenous, and entirely separated from the epiphysis, the latter remaining in most cases healthy. This last condition may be explained by the peculiar disposition of the periosteum at the epiphysial line. The membrane here divides into two processes, a stronger and thicker one, which passes in to be attached to the cartilage at the epiphysial line: and a thinner one which is continued down towards the joint. This anatomical arrangement explains the frequent escape of the epiphysis in acute disease of the shaft. When extension however takes place to the joint, the line of inflammation is generally through the middle of the epiphysis, and very rarely along its surface. In less severe cases of acute periostitis a portion of the shaft generally towards the centre becomes necrosed. The cause of the escape of the distal parts of the shaft is the comparatively rich blood supply it receives from vessels, which pass into it from the epiphysis through the growing line.

But if we are to explain satisfactorily the exceedingly rapid onset of the disease, the concurrence of periostitis and osteomyelitis—and specimens examined shortly after removal show both conditions to be present—the early and complete separation of the shaft with necrosis of its whole thickness: some region of bone other than the periosteum or medulla must be the site of earliest inflammation. This region I have experimentally shown to be the juxta-epiphysial junction; inflammation commencing here spreads to the medulla and outwards to the periosteum.

In some experiments on rabbits carried out at the Pathological Institute of Leipzic I found that if an incision were made in the lower end of the left femur and the bone drilled, and some sterilised distilled water containing pure culture of staphylococci pyogenes aurei inserted, and the wound closed, the animal died in twenty-six to thirty hours afterwards. On *post-mortem* examination the medulla of the femur was very red and semi-fluid, the periosteum entirely unaffected, but at the juxta-epiphysial junction there were numerous semi-purulent foci. In other experiments, on inserting a dose of staphylococci into the lumen of the external jugular vein, death ensued in four to six days. There were found numerous collections of pus in the viscera; but on examining the various bones in no instance was there pus beneath the periosteum. The medulla of several bones was much injected, darkish and patchy, but there was no pus present. In the juxta-epiphysial junction several purulent foci were found. Still further, if the periosteum in another animal were carefully separated for a considerable extent off the bone, and then beneath the periosteum a mass of staphylococci from a pure culture were inserted and the parts replaced as carefully as possible, on death ensuing no pus was found beneath the periosteum, but both medulla and juxta-epiphysial line showed marked inflammatory changes.

From these experiments one is able to affirm with much show of reason that the periosteum is less susceptible to the influence of micro-organisms than the other two regions above named. Placing these experimental results side by side with the morbid appearances seen in severe cases where there is actual loss of tissue at the ends of the shaft, the conclusion naturally follows that the disease commences earliest in the juxta-epiphysial region, and is always furthest advanced here. A more precise and better term, therefore, than acute periostitis is acute juxta-epiphysitis.

The symptoms of the disease are typically as follows: The patient, in most cases a boy about the age of puberty, receives a blow, for example, on the tibia. He takes no notice of it for two or three days, and is then seized with very severe rigors; the temperature rapidly reaches a high point, frequently touching 104 degrees. Severe pain ensues, often affecting the whole limb. In the earliest stages, the veins of the leg are distended, and the soft tissues near the affected spot are swollen. Within a few hours deep fluctuation may be detected, if the tension of the effused fluid is not too great. Later, much redness and inflammatory œdema are noticed; and when the periosteum has burst, fluctuation is easily obtained. With these local signs the gravity of the general symptoms deepens, and there may be delirium or coma. So much so, that the disease has been mistaken for enterica, meningitis, or ulcerative endocarditis. The most common error is to mistake it for acute rheumatism.

*Treatment.*—Influenced no doubt by the belief that they are dealing merely in the first instance with acute periostitis, surgeons have often been content merely to incise the periosteum. But the insufficiency of this mode of treatment is frequently shown by the subsequent course of the disease, in which, notwithstanding incision, death of the shaft has taken place and general pyæmia resulted. I would urge most strongly that even in the early stage of the disease, when there is marked pain associated with swelling and fulness of the veins of the limb and rigors, it is essential, in addition to incising the periosteum, to tunnel into the bone and open up the medullary cavity. This should be done in two places, in the juxta-epiphysial region and at a point about an inch higher up, and so open up at once all three structures, the periosteum, the juxta-epiphysial junction, and the medulla. Should improvement not then set in, it is not only permissible, but rational to open up the medullary cavity at the other extremity of the diaphysis, and clear out the inflamed marrow.* Such a course can have no possible effect on the subsequent growth of the bone, so long as the aperture is not made through the epiphysial line. Its early adoption can only be attended with such good results that removal of the entire shaft either by sub-periosteal resection will no longer be called for; the neighbouring joint is not likely to be affected by extension through the epiphysis; and the patient will be spared not only the immediate dangers of pyæmia, but the more prolonged ones arising from long-continued suppuration, due to exfoliation of the necrosed bone with formation of new bone.

